# Rapid Gene Family Evolution of a Nematode Sperm Protein Despite Sequence Hyper-conservation

**DOI:** 10.1534/g3.117.300281

**Published:** 2017-11-21

**Authors:** Katja R. Kasimatis, Patrick C. Phillips

**Affiliations:** Institute of Ecology and Evolution, University of Oregon, Eugene, Oregon 97403

**Keywords:** molecular evolution, gene family evolution, reproductive proteins, nematodes

## Abstract

Reproductive proteins are often observed to be the most rapidly evolving elements within eukaryotic genomes. The major sperm protein (MSP) is unique to the phylum Nematoda and is required for proper sperm locomotion and fertilization. Here, we annotate the MSP gene family and analyze their molecular evolution in 10 representative species across Nematoda. We show that MSPs are hyper-conserved across the phylum, having maintained an amino acid sequence identity of 83.5–97.7% for over 500 million years. This extremely slow rate of evolution makes MSPs some of the most highly conserved genes yet identified. However, at the gene family level, we show hyper-variability in both gene copy number and genomic position within species, suggesting rapid, lineage-specific gene family evolution. Additionally, we find evidence that extensive gene conversion contributes to the maintenance of sequence identity within chromosome-level clusters of MSP genes. Thus, while not conforming to the standard expectation for the evolution of reproductive proteins, our analysis of the molecular evolution of the MSP gene family is nonetheless consistent with the widely repeatable observation that reproductive proteins evolve rapidly, in this case in terms of the genomic properties of gene structure, copy number, and genomic organization. This unusual evolutionary pattern is likely generated by strong pleiotropic constraints acting on these genes at the sequence level, balanced against expansion at the level of the whole gene family.

Postinsemination reproductive tract dynamics are fundamentally important for determining an individual’s reproductive success. In animals with internal fertilization, the male ejaculate must interact with the female reproductive tract and ovum, as well as potentially needing to outcompete the sperm of other males. Just as preinsemination processes are shaped by sexual selection, so too are postinsemination interactions. However, the dynamics of the latter case are predominantly driven by molecular interactions, as opposed to behavioral ones, and therefore the appropriate unit of evolutionary analysis is the molecular evolution of the reproductive proteome (McDonough *et al.* 2016; Wilburn and Swanson 2016). Studies across a wide range of vertebrate and invertebrate taxa have consistently shown that reproductive proteins have an elevated ratio of nonsynonymous to synonymous substitutions relative to nonreproductive proteins (Swanson and Vacquier 2002; Clark *et al.* 2006; Vacquier and Swanson 2011; Mordhorst *et al.* 2015). In fact, sperm-specific, seminal fluid, and egg-specific proteins evolve at astonishingly rapid rates, and are often the fastest observed within a given genome. Within these reproductive categories, evolutionary rates differ based on sex and functional protein class. Specifically, male reproductive proteins evolve more rapidly than their female counterparts (Drosophila 12 Genomes Consortium *et al.* 2007; Harrison *et al.* 2015) and, within male proteins, seminal fluid proteins show the strongest signals of positive selection (Begun *et al.* 2000; Wagstaff 2005; Drosophila 12 Genomes Consortium *et al.* 2007; Findlay *et al.* 2009; Walters and Harrison 2010; Dean *et al.* 2011). These rapid evolutionary rates in males are often attributed to sexual selection in the form of sperm competition (Dhole and Servedio 2014). However, male reproductive proteins are involved in a variety of roles including sperm motility, antimicrobial response, oxidative protection, sperm capacitation, and immunity modulation, in addition to modifying female behavior and physiology (Poiani 2006; Perry and Rowe 2015). Such a diversity of functions suggests that pleiotropic trade-offs may be common and that these signatures of protein evolution may in fact be driven by multiple selective pressures (Poiani 2006; Good *et al.* 2013; Dapper and Wade 2016).

The standard approach to studying reproductive proteins is gene-based: the sequence evolution of a gene of interest is analyzed across multiple species. While this approach provides valuable information, it does not capture the full effects of selection across the levels of genomic organization. In particular, gene families are highly dynamic in their genomic organization, gene copy number, and transcriptional architecture (Demuth and Hahn 2009; Innan and Kondrashov 2010; Schrider and Hahn 2010), creating an additional source of variation upon which selection can act (Perry *et al.* 2007; Xue *et al.* 2008; Conrad *et al.* 2010). For example, positive selection can drive gene family expansion through selection for divergent gene copies or maintain neutrally duplicated genes (Innan and Kondrashov 2010). Therefore, to fully understand the evolutionary history of a gene, both genic and genomic approaches are necessary to capture the multiple levels of genomic organization.

Nematodes are an excellent system for taking a genomic-based approach to reproductive protein evolution and addressing standing questions on the pleiotropic trade-offs influencing their evolution. First, multiple annotated reference genomes exist (Blaxter and Koutsovoulos 2015), which allows gene families to be analyzed for both structure and organization. Additionally, nematodes exhibit variation in life history (Blaxter and Koutsovoulos 2015), including the presence of multiple mating systems (Felix *et al.* 2014)—gonochoristic and self-fertilizing hermaphroditic—creating variation in the mechanisms influencing mating and sperm dynamics. Finally, nematodes have a unique sperm biology characterized by large, crawling sperm (Justine 2002). The most abundant protein family is the MSP (Klass and Hirsh 1981; Burke and Ward 1983). This multi-gene family has almost exclusively been described biochemically (Burke and Ward 1983; Haaf *et al.* 1998; Smith and Ward 1998; Baker *et al.* 2002). Specifically, MSP is a dimeric molecule that polymerizes to form branching filaments, which form the pseudopod of the cell and are used to crawl in a treadmilling fashion (Burke and Ward 1983; Bottino *et al.* 2002; del Castillo-Olivares and Smith 2008). These filaments are structurally similar to actin filaments and, in fact, MSP replaces the function of actin in sperm cells (Nelson *et al.* 1982). In addition to its role in locomotion, studies in *Caenorhabditis elegans* have shown that MSP has pleiotropic effects, namely acting as an oocyte signaling molecule (Miller *et al.* 2001). Despite their central role in fertilization, MSP genes have not been rigorously annotated outside of *C. elegans*, nor has the molecular evolution of this gene family been characterized.

Here, using a novel annotation of the large MSP gene family across 10 different species, combined with rate-based tests and an analysis of synteny, we show that MSPs display a remarkable combination of nearly complete sequence conservation at the individual sequence level contrasted with extensive lineage-specific evolution of the gene family within species. Thus, nematode MSPs appear to be yet another example of the rapid evolution of reproductive proteins, but in this case, this pattern emerges only when the entire genomic context of the gene family is taken into account.

## Materials and Methods

### MSP gene annotations

The *C. elegans* MSP gene family (PRJNA13758) was used as the reference sequence for annotations. The *C. elegans* genome is a high-quality whole-genome assembly (CEGMA: 100% complete, 0% partial and BUSCO 98% complete, *n* = 982) (Howe *et al.* 2017) with well-curated annotations (Lee *et al.* 2017), and therefore we are confident using the annotated MSP genes as our query data set. Thirty-one MSP genes have been identified, predominately using biochemical and molecular genetic techniques (Burke and Ward 1983). Note that the gene sequence for *msp-32* is markedly different from the other *C. elegans* MSP genes in overall length, so we verified the predicted sequence using PCR amplification of the gene from the standard N2 lab reference strain and Sanger sequencing.

MSP genes were annotated in the genomes of nine species: *C. sp. 34* (PRJDB5687), *C. briggsae* (PRJNA10731), *C. remanei* (PRJNA248909), *C. angaria* (PRJNA51225), *Pristionchus pacificus* (PRJNA12644), *Strongyloides stercoralis* (PRJEB528), *Ascaris suum* (PRJNA62057), *Wuchereria bancrofti* (PRJNA275548), and *Trichinella spiralis* (PRJNA257433). Annotations were made using custom blast searches in Geneious v9.1.5 (Kearse *et al.* 2012). Blast searches were conducted using all 31 *C. elegans* MSP gene copies based on nucleotide sequence (Megablast) for *Caenorhabditis* species, and amino acid sequence (tblastn) for the other species. Results were hand-curated to ensure accuracy in assignment and predicted gene annotations. Specifically, all blast results were checked to ensure that the hit corresponded to a true gene (*i.e.*, contained a start and stop codon) and contained an MSP domain (Tarr and Scott 2005). A total of 121 genes were annotated across the nine species. The predicted gene annotation was edited in five genes due to a miscalled start or stop codon, or a miscalled intron splice site.

MSP secondary structure was predicted using the Phyre^2^ server (Mezulis *et al.* 2015). Structural models and residue mapping were visualized using the PyMOL Molecular Graphics System v1.8 (Schrödinger, LLC).

### Evolutionary rate tests

The MSP gene sequences were aligned using ClustalW (Thompson *et al.* 1994). Amino acid divergence of the global sequence alignments was calculated for all pairwise gene combinations within a species. Because the unusual nature of evolution in this gene family precluded orthology assignments across family members, we also calculated the distribution of pairwise divergences relative to the *C. elegans* reference rather than attempting to estimate phylogeny-based measures of the average rate of evolutionary change, such as ω (Yang 2007). Unrooted maximum likelihood phylogenies were constructed in PhyML based on sequence alignments of all genes across all species (Guindon and Gascuel 2003). To corroborate that the MSP genes on chromosome II form species-specific clades based on chromosome-level clustering, we calculated the approximate likelihood of the inferred topology relative to the next most likely tree without species-specific clades (Supplemental Material, File S1) (Anisimova and Gascuel 2006). The test was run against five independently inferred, randomized phylogenies to avoid being caught in a local maximum.

To determine if nucleotide sequence identities were higher within genomic clusters than between clusters, we conducted a permutation analysis of pairwise sequence identity by randomizing the order genes throughout the genome and computing the difference in mean nucleotide sequence identity of the randomly reassigned clusters, using clusters of the same size of those observed within the genome. This allowed us to create a null distribution in which the hypothesis that sequence identity did not depend on genomic location was true (difference between measures equal to zero). This distribution was generated from a total of 10^5^ permutations, and the probability of rejecting the null hypothesis was calculated by examining how often the randomized comparisons equaled or exceeded the observed difference among the actual clusters.

### Synteny analyses

Synteny of the MSP genes within *Caenorhabditis* was analyzed using species with high-quality whole-genome assemblies: *C. elegans*, *C. sp. 34*, *C. briggsae*, and *C. remanei*. The *C. elegans* MSP genes form three gene clusters: one on chromosome II and two on chromosome IV. Additional genes falling within these clusters that were able to serve as syntenic chromosome anchors were identified using the UCSC Genome Browser (Kent *et al.* 2002). The chromosome II gene anchors were highly conserved across these species, and were located on the chromosome or scaffolds to which MSP genes also mapped (Table S1 in File S2). The chromosome IV gene anchors displayed more variation in the location to which they mapped across species, and had little to no overlap with the MSP genes annotated in these species (Table S2 in File S2). Therefore, only the MSP genes that mapped to chromosome II were included in the synteny analyses and all the other MSP genes were categorized as unique to their given species.

### Gene dosage analyses

To determine if gene copy number was correlated with gene dosage, we performed a linear model of copy number *vs.* sperm size within R v3.2.1 (R Core Development Team 2015). Sperm size—given as spermatid diameter—was obtained from estimates provided in the literature: *C. elegans* (Vielle *et al.* 2016), *C. sp. 34* (Woodruff *et al.* 2017), *C. remanei* (Vielle *et al.* 2016), *C. briggsae* (Vielle *et al.* 2016), *C. angaria* (Vielle *et al.* 2016), *P. pacificus* (Rudel *et al.* 2005), and *A. suum* (Theriot 1996).

As an additional test of the possible influence of gene dosage and gene family diversity, gene-expression patterns were analyzed for *C. elegans* using median expression within larval stage four males, as assembled within WormBase (Lee *et al.* 2017). We fitted a linear model to determine if either (i) chromosome-level clusters or (ii) isopeptide subfamilies predicted MSP expression patterns.

### Data availability

All data used are publicly available as outlined above. The code used to generate approximate likelihood ratios for the gene trees is available in File S1.

## Results

### MSP gene family annotation

**Figure 1:**
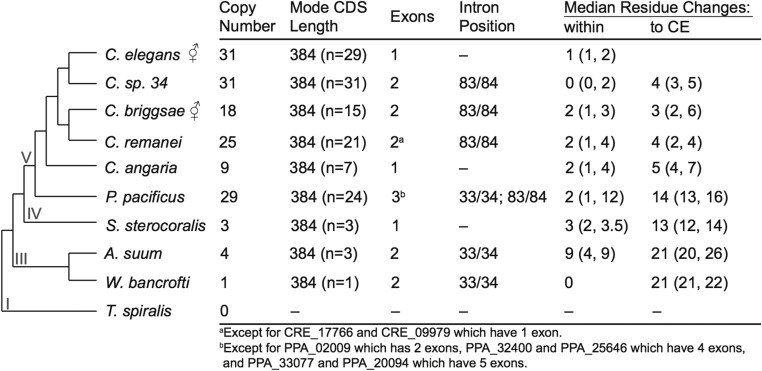
The evolution of the major sperm protein (MSP) gene family across Nematoda [species tree from Blaxter and Koutsovoulos (2015)]. For each species, the number of gene copies, coding sequence length (CDS; given as the mode), number of exons, amino acid residues between which the intron(s) is located, and sequence divergence estimates are given. Sequence divergence is given as the median number of pairwise amino acid residue changes within MSP gene copies of each species, as well as the pairwise divergence between the copies of each species and the 31 *C. elegans* (CE) reference MSP genes. The lower and upper quartiles of the pairwise divergences are given in parentheses. Species from the basal nematode clades have fewer MSP gene copies relative to clade V species. However, there is a high degree of sequence conservation across all species. The estimated evolutionary divergence time within *Caenorhabditis* is tens of millions of years, while the common ancestor between CE and clade III is estimated to have diverged over 500 million years ago (Blaxter 2009).

We annotated MSP genes in nine representative species across Nematoda using the 31 *C. elegans* MSP gene copies as a reference (Figure 1). Species were chosen from four of the five major nematode clades (Blaxter and Koutsovoulos 2015) based on the availability of high-quality whole-genome assemblies. We sampled five species from the *Caenorhabditis* genus to capture variation across different mating systems and to provide the context for fine-scale genomic analysis. For each of the species chosen, we blasted each of the *C. elegans* MSP genes against the reference genome. We annotated MSP genes in eight of the nine species. Interestingly, we were unable to annotate any MSP genes in *T. spiralis* (clade I). The amino acid sequence identity of potential *T. spiralis* orthologs to the *C. elegans* gene family was at most 37.5% identical (T01_10172), with no identifiable MSP-domain motifs, so we expanded the blast search to include all the MSP genes annotated in the other eight species. Again, we did not find amino acid sequence identity >39.2% (exon 3 of T01_1333 to *P. pacificus*). The genus *Trichinella* is reported to have crawling sperm (Justine 2002) and therefore the complete lack of MSP genes seems unlikely. If very few gene copies are present, as may be the case due to the global decrease in genes in the lineage leading to *T. spiralis* (Markov *et al.* 2014), then the sequence could simply be missing from the genomic information available, despite the high quality of the genome (CEGMA: 96.8% complete and 0.0% partial, and BUSCO: 87.4% complete for *n* = 982). In contrast to the apparent lack of MSP in *T. spiralis*, we identified four MSP genes in *A. suum*, contrary to biochemical-based reports of a single gene with two isoforms (King *et al.* 1992).

In the nematode genomes with clearly identifiable MSP genes, copy number ranged from 1 to 31 (Figure 1). Gene copy number appears to have dramatically increased in the clade V nematodes. This copy number increase may be a general pattern across clade V species [see Markov *et al.* (2014)] or could potentially be an artifact of the genomes available. Currently only high-quality genomes exist for parasitic species for nonclade V nematodes, while clade V genomes all come from free-living species. Parasitism can lead to reductions in genome size Hunt *et al.* (2016) and, while there is no specific evidence for overall genome reduction in these nematodes, fewer coding genes are annotated in these parasitic species relative to free-living ones (Howe *et al.* 2017). Alternatively, increases in gene copy number are often associated with selection for increasing gene dosage (Ohno 1970). If true, sperm size and MSP gene copy number would be predicted to be positively correlated, as larger cells would require more protein to move (Burke and Ward 1983). In contrast, we did not find a correlation between sperm diameter and gene copy number (F_1,5_ = 0.80 and *P* = 0.41). Nor was there an apparent trend between mating system (hermaphroditic or gonochoristic) and gene copy number.

Coding-sequence length was conserved across the phylum (mode CDS length = 384 nt for 134 of 152 gene copies annotated). However, the number of exons varied between species, though within a species the number of exons and the intron splice site was conserved (except for five genes in *P. pacificus* and two genes in *C. remanei*, Figure 1). A parsimonious model of intron evolution suggests an ancestral gene state of two exons with a single, short intron toward the beginning of the gene. In the lineage leading to clade V, there appears to have been a gain of a second intron toward the end of the gene, with a secondary loss of the ancestral intron position within the lineage leading to *Caenorhabditis*. In *P. pacificus*, MSP genes had both a greater number of exons and more variability in the number of exons than seen in the other species, consistent with previous studies (Rödelsperger *et al.* 2013). Three of the species sampled—*S. stercoralis*, *C. angaria*, and *C. elegans*—showed independent losses of introns in all gene copies.

### The MSP amino acid sequence is hyper-conserved

Given the two very different functions of the MSP during postinsemination dynamics—locomotion and signaling—we expected to see patterns that might reflect the evolutionary divergence of protein function. The median amino acid divergence between MSP gene copies within a species was <2.5% for all species except *A. suum*, which had a median within-species divergence of 7% (Figure 1). These low within-species divergences suggested that the MSP amino acid sequence has been highly conserved within individual lineages. Comparisons of sequence divergence across the phylum revealed that the median pairwise divergence for each species compared to *C. elegans* ranged from 2.3 to 16.5%, with sequence divergence increasing with evolutionary distance. In particular, the maximum median amino acid divergence (16.5%) was seen between *C. elegans* and both clade III representatives, representing over a billion years of total evolutionary divergence time (Blaxter 2009). This extremely low level of sequence divergence is comparable to known highly conserved, ancient gene families such as actin (Mills *et al.* 2001), histone (Pehrson and Fuji 1998; Malik and Henikoff 2003), and ubiquitin (Sharp and Li 1987; Tan *et al.* 1993). For example, mouse and human actin homologs have 79–88% sequence identity (Mills *et al.* 2001). In comparison, the degree of genomic divergence between mouse and human is roughly similar to that between *C. elegans* and *C. briggsae* (Kiontke 2005), which have a mean MSP sequence identity of 95%. In order to perform a direct evolutionary rate comparison to determine the extent of MSP sequence conservation, we calculated the amino acid divergence for the actin gene family within *Caenorhabditis*. The median pairwise divergence of actin paralogs across *Caenorhabditis* species ranged from 0.8 to 1.1% (Table S3 in File S2). These actin divergence values are very comparable to those seen within MSP gene copies of each of the *Caenorhabditis* species (median within-species pairwise divergence range: 0–1.5%), while divergence among species was slightly higher (median pairwise divergence to *C. elegans* range: 2.3–3.9%). Conversely to what is seen in actin, MSP sequence conservation appears to be stronger within a species than divergence among species, potentially due to the young age of paralogs or strong within-species constraint. Overall, within *Caenorhabditis*, the MSP genes appear to evolve at a rate similar to actin, making this one of the most highly conserved gene families known.

**Figure 2:**
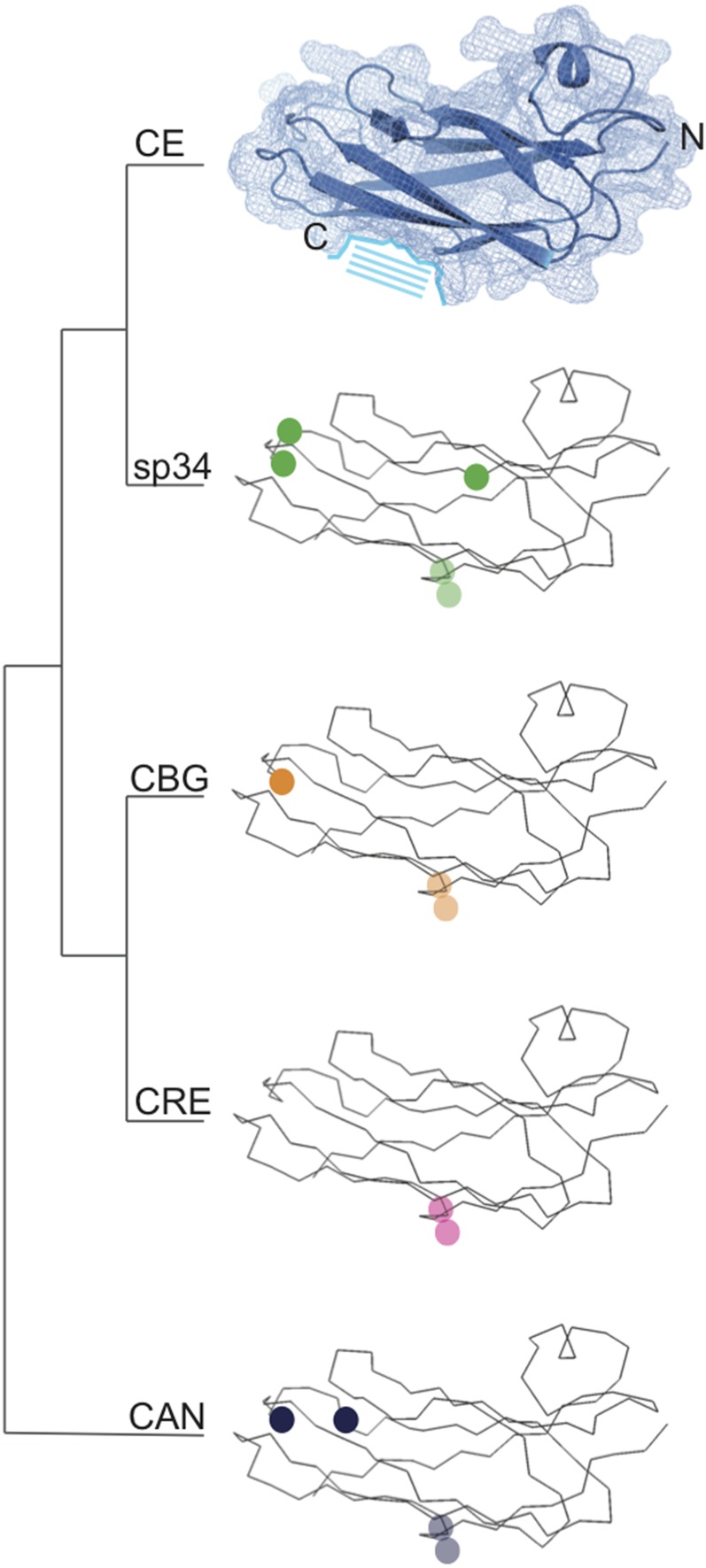
The major sperm protein (MSP) amino acid sequence is highly conserved across *Caenorhabditis*. A space-filling molecule highlighting secondary structure is shown for *C. elegans* (CE). The N-terminus (N), C-terminus (C), and dimer interface (highlighted with stripes) are shown. Ribbon structures are shown for *C. sp. 34* (sp34), *C. briggsae* (CBG), *C. remanei* (CRE), and *C. angaria* (CAN). Circles mark amino acid changes relative to CE present in three or more species-specific isopeptide subfamilies (Table S4 in File S2). Transparency is used for the residues on the back side of the molecule. The residue changes highlighted are consistent across species and do not fall in predicted binding domains. Protein structures were obtained from the Phyre^2^ server (Mezulis *et al.* 2015) using the published MSP crystal structure (Baker *et al.* 2002).

The low within-species amino acid divergence of MSPs in the *Caenorhabditis* species is primarily caused by multiple genes having invariant protein sequences. These protein sequence identities allowed us to group MSP genes into species-specific subfamilies based on isopeptide sequence (Figure S1 and Table S4 in File S2). Even after grouping redundant sequences, most subfamilies had no more than five amino acid residues that were different from the *C. elegans* reference (Figure 2). Further, the majority of amino acid changes at any given residue occurred in only a single subfamily rather than across all subfamilies of a species (Figure 2 and Figure S1). Three residues in particular (15G, 16T, and 80F) appear less constrained than the rest of the amino acid sequence (Figure 2). These residues are not involved in protein folding or filament formation (Haaf *et al.* 1998; Baker *et al.* 2002; del Castillo-Olivares and Smith 2008), suggesting that they likely do not affect locomotion. Additionally, there were very few amino acid changes in the end of the protein sequence (residues 109–127). These residues have been shown to be essential for both filament formation (del Castillo-Olivares and Smith 2008) and the stimulation of oocyte release (Miller *et al.* 2001), highlighting the strong functional constraint on the amino acid sequence. A noticeable exception to this strong whole-protein sequence conservation was seen for four genes that each comprise a unique subfamily. These subfamilies had a diverged end located at either the N-terminus (CE-13) or C-terminus (sp34-4, CBG-1, and CBG-2) (Figure S1). These diverged termini range from 19 to 113 amino acids in length and have no predicted secondary structure. Given the high degree of sequence similarity in the rest of the protein, these additional domains are unexpected and may represent MSP proteins with functions outside of locomotion, although their actual function is currently unknown.

### Lineage-specific MSP gene family evolution within Caenorhabditis

We took advantage of the high MSP copy number within *Caenorhabditis* to explore the evolutionary history of the MSP gene family from a genomic perspective. Due to the high degree of sequence conservation, we could not rely on traditional sequence-based approaches [such as Yang (2007)] to infer evolutionary homology. Therefore, we instead took a synteny-based approach coupled with phylogenetic relationships structured by synonymous variation to examine orthology. Specifically, if the MSP gene family was a large, ancestral family, we expected to see: (1) conservation of synteny across species and (2) phylogenetic clustering of orthologous gene copies from each species into monophyletic clades.

**Figure 3:**
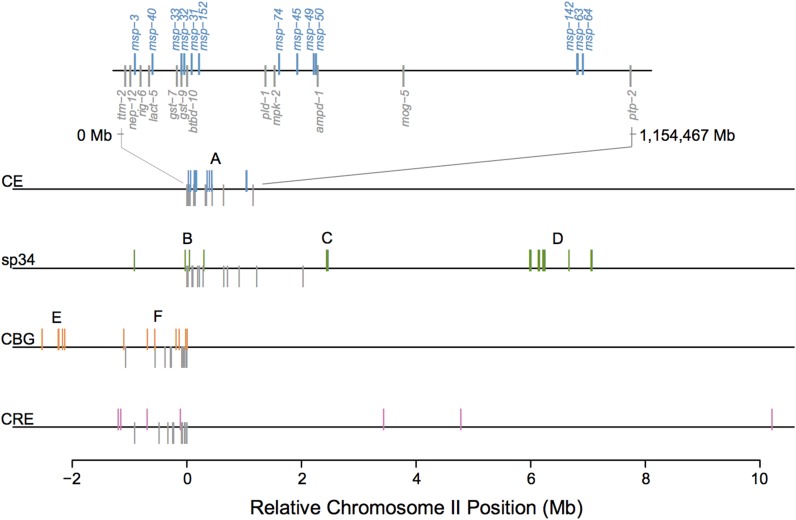
Major sperm protein (MSP) genes are not syntenic across *C. elegans* (CE), *C. sp. 34* (sp34), *C. briggsae* (CBG), and *C. remanei* (CRE). The majority of MSP genes map to chromosome II. The syntenic region is defined around the CE gene anchors (shown as a gray downstrike, Table S1 in File S2). The *x*-axis is given as relative chromosome II position, which was defined by setting the first gene anchor (*ttm-2*) as the origin. These anchors are conserved and syntenic across species, although they are in an inverted orientation in *C. briggsae* and *C. remanei*. The MSP genes in sp34 (green upstrike), CBG (orange upstrike), and CRE (pink upstrike) do not fall within the gene anchors, but rather form nonsyntenic clusters across the chromosome. The MSP gene cluster labels correspond to the phylogenetic clades labeled in Figure 4.

Chromosome II was the only genomic location in which *C. elegans*, *C. sp. 34*, *C. briggsae*, and *C. remanei* had overlapping occupancy of MSP genes (Table S1 and Table S2 in File S2). *C. angaria* was not included due to incomplete genome assembly in this region. We used a conserved set of 12 genes on chromosome II, spanning the *C. elegans* chromosome II MSP gene cluster, to provide a genomic scaffold against which to evaluate the local evolution of MSP genes (Table S1 in File S2). The gene anchors were conserved and syntenic between *C. elegans* and *C. sp. 34* (Figure 3). The order of the anchors was also conserved in *C. briggsae* and *C. remanei* but in an inverted orientation. Importantly, the MSP genes form separate gene clusters across the chromosome that are distinct within each species, with little overlap relative to the gene anchors. Additionally, all the species had MSP gene clusters on chromosome II that occupied regions in which MSP genes are completely absent in *C. elegans*, and each within-species gene cluster occupied a unique region of chromosome II.

**Figure 4:**
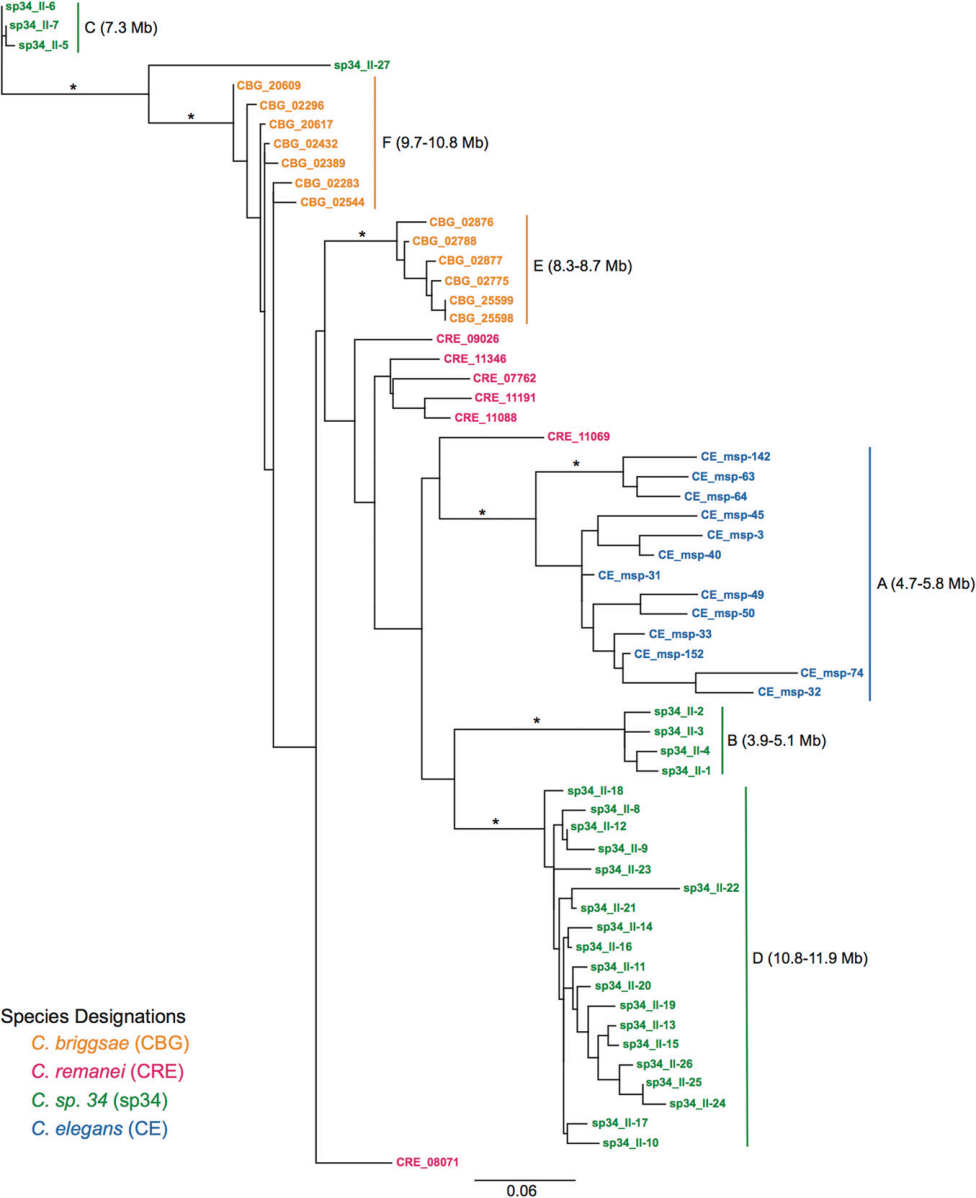
Maximum likelihood phylogeny of the major sperm protein (MSP) genes that map to chromosome II. The labeled monophyletic clades correspond to distinct chromosome-level MSP gene clusters (shown in Figure 3). Asterisks denote bootstrap values >80%. Approximate likelihood ratio analysis supports this topology as the best representation of the evolutionary relationships (mean log-likelihood = −3910.59 for five independently inferred phylogenies). The structuring of phylogenetic variation based on gene clusters and complete lack of recapitulation of species relationships suggests that the MSP genes are not orthologous.

**Figure 5:**
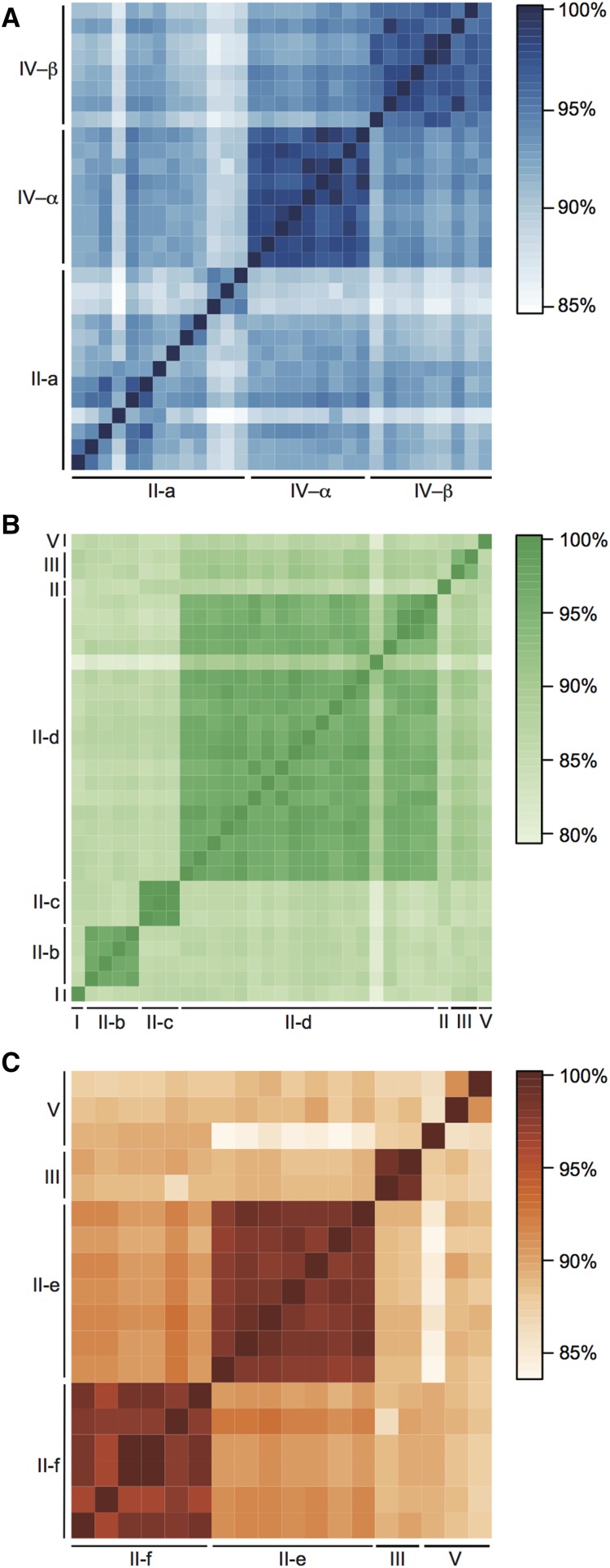
Nucleotide sequence identity for (A) *C. elegans*, (B) *C. sp. 34*, and (C) *C. briggsae*. Each square represents the percent sequence identity between a gene pair. The genes are ordered increasing along each chromosome from I to V, as applicable. The majority of major sperm proteins (MSPs) map to chromosome II in all species. These MSPs are labeled based on their chromosome-level cluster (II-a through -f), corresponding to the labels in Figure 3 and Figure 4. Overall, genes are more similar within these chromosome-level clusters than between clusters in all three species (*P* < 0.0001).

Despite the homology of MSP genes and some overlap of genes with the syntenic chromosome II anchors, phylogenetic analysis did not show one-to-one MSP orthologs across species. Rather, phylogenetic structuring of chromosome II MSP genes mirrored the physical grouping of genes, such that monophyletic clades corresponded to each species-specific MSP chromosome-level gene cluster (log-likelihood of species-specific clades = −3910.59, Figure 4). Indeed, local monophyletic structure within clusters was maintained when all gene copies in the genome were included in the analysis (Figure S2). Further, phylogenetic analysis of only the MSP clusters overlapping with the chromosome II syntenic anchors (Figure 4, clusters: CE-A, sp34-B, and CBG-F) reinforced species-specific monophyly (data not shown). The strict structuring of predominantly synonymous nucleotide variation within gene clusters is contrary to an expectation of local syntenic identity-by-descent and lacks concordance with known species relationships. Instead, gene sequence history appears to track genes through cluster-specific gene conversion via nonhomologous DNA repair (Innan and Kondrashov 2010). The role of gene conversion appears to be particularly strong when examining within-species pairwise nucleotide sequence identities across the whole genome (Figure 5). As seen in *C. elegans*, *C. briggsae*, and *C. sp. 34*, nucleotide variation—and, in particular, synonymous variation—is more similar within genomic clusters than between clusters (*P* < 0.001). Additionally, within gene clusters, the physical proximity of genes appears correlated with sequence identity, as seen for the *C. elegans* chromosome II cluster (Figure 5A), further supporting the action of gene conversion. This pattern of unique, nonsyntenic gene clustering at both the physical chromosome and evolutionary history levels does not support the expectation of an ancestral, preserved gene family. Rather, such a pattern is reflective of a model in which gene copy variation is generated by lineage-specific duplications, with sequence identity enforced within tandem duplicates by cluster-specific gene conversion.

### Patterns of expression do not explain gene family evolution within C. elegans

Within *C. elegans*, we were able to directly assess MSP gene expression, and examine the relationship between expression and genomic organization and sequence hyper-conservation. Specifically, using RNA expression data, we examined if chromosome-level clustering or isopeptide subfamily designation were correlated with gene-expression patterns. Gene-expression differences between chromosome-level clusters were marginally significant (F_2,28_ = 4.99 and *P* = 0.014), with cluster IV-β having the highest mean expression and IV-α the lowest (mean expression and SE for chromosome II: 2c343.4 ± 423 FPKM, chromosome IV-α: 1,263.3 ± 276 FPKM, and chromosome IV-β: 3150.5 ± 406). Perhaps more importantly, expression within an individual cluster could range by an order of magnitude in adjacent genes. Gene expression differences among isopeptide subfamilies were also marginally significantly different (F_12,18_ = 2.36 and *P* = 0.048). Interestingly, *msp-32*, a diverged terminus MSP, had the lowest expression, though again the functional implications require more targeted information.

## Discussion

Male reproductive proteins have come to be synonymous with rapid evolution driven by sperm competition and antagonistic male–female coevolution (Swanson and Vacquier 2002; Wilburn and Swanson 2016). Here, combining custom annotation of MSP genes with genic and genomic analyses, we investigated the evolutionary history of the MSP gene family across the phylum Nematoda. The MSP is arguably the most important nematode sperm protein and, given our knowledge of sperm protein evolution in other systems, we expected to see signatures of positive selection. However, MSPs do not conform to this standard expectation. Rather, these genes show a degree of hyper-conservation that is observed in fundamental eukaryotic proteins, such as actin. Specifically, >83.5% amino acid sequence identity has been maintained for the >500 million years since these groups shared a common ancestor, making MSP genes some of the most conserved genomic elements yet identified.

The high degree of constraint observed is potentially reflective of the pleiotropic trade-offs to which MSP genes are subject. Biochemical studies of MSP have identified that much of the protein is important for proper dimerization and filament formation (Haaf *et al.* 1998; Baker *et al.* 2002; del Castillo-Olivares and Smith 2008). Further, nonsynonymous mutations at these interaction sites result in incorrect or loss of filament formation (del Castillo-Olivares and Smith 2008). Such strong functional constraint likely results in equally strong purifying selection, as mutations of this sort could effectively poison a cell through the loss of locomotory function and therefore prevent fertilization from being achieved. Thus, given these structural dependencies and their fundamental role in the most basic attribute of fitness, fertilization, it is perhaps not surprising that MSPs are highly constrained (albeit at nearly every single amino acid). However, the MSP also acts as an oocyte signaling molecule. Here, we would predict that we would see sexual selection resulting from male–female dynamics drive sequence divergence of gene copies. Four genes had a diverged terminus, possibly reflective of such neofunctionalization, and further functional characterization of these genes is warranted. Nevertheless, within a species MSP copies are essentially identical, suggesting that strong pleiotropic trade-offs can hinder evolution driven by intersexual interactions.

While well studied in other contexts, gene family dynamics are still underappreciated in reproductive protein studies. We found evidence of extensive MSP gene family evolution within *Caenorhabditis* in the face of the strong pleiotropic constraint on gene sequence variation. Two alternative models can explain the emergence of dynamic gene family variation across a genus. First, a large set of paralogs could be derived from a common ancestor with subsequent differentiation within each lineage. Alternatively, there could be lineage-specific evolution, such that the gene copies arose after branching from a common ancestor and are therefore unique to each lineage. Our data best support a lineage-specific model of gene family evolution, whereby the MSP gene family evolves through independent gene translocations, followed by tandem duplication and cluster conservation via gene conversion (Figure 3 and Figure 4). Three lines of evidence indicate this model of evolution: synteny analysis, phylogenetic structuring of synonymous variation, and intron evolution. MSP genes form distinct, species-specific clusters across the genome that are highly variable in both the number of genes present and the physical length of chromosome occupied. If clusters of MSP genes were preserved from an ancestral family and subsequently translocated as clusters throughout the genome, we would expect to see proportional spacing of MSP genes through clusters with simultaneous translocation of linked genes. Instead, syntenic analysis provides no evidence of gene hitchhiking within clusters. Rather, these data support independent movement of single genes throughout the genome. A pattern of tandem gene duplication follows a translocation event, which is supported by the phylogenetic grouping of gene clusters based on synonymous nucleotide variation. Further, there is a lack of recapitulation of known species relationships within the gene trees, again suggesting independent duplication events. These phylogenetic patterns also suggest strong gene conversion within MSP gene clusters as the mechanism by which sequence identity is maintained. Gene conversion was particularly evident in the extremely high sequence similarity of synonymous variation within genomic clusters, while more variation was measured between clusters (Figure 5). While gene conversion can mask signals of orthology, we do not believe this to be the case. In particular, the patterns of intron loss observed are not consistent with the maintenance of ancestral paralogs, as it is highly unlikely that a conserved family would lose all introns simultaneously across the genome. Rather, MSP genes appear to have a highly dynamic nature that is independent within each *Caenorhabditis* species. While this pattern of sequence conservation and gene family evolution is not unique to the MSP family [see Perry *et al.* (2007), Sackton *et al.* (2007), Gao and Zhu (2016), and Lee *et al.* (2016)], the degree of copy number variation and genomic reorganization seen for the MSP family is more extensive than previously observed.

Lineage-specific duplications have been quantified on a broad scale across Nematoda and are believed to be related to dosage constraints (Markov *et al.* 2014; Baskaran *et al.* 2015) and life history transitions (Baskaran *et al.* 2017). However, the mechanism driving this rapid lineage-specific evolution within a single genus is still somewhat unclear. Gene families can be positively selected for diversification of gene copies, which is clearly not the case for the MSP gene family since the amino acid sequence is highly conserved both within and between species (Figure 1). Positive selection can also act to change the transcriptional architecture of a family and thereby affect gene dosage (Innan and Kondrashov 2010). Again, this mechanism does not appear to drive MSP gene family evolution, as gene copy number is decoupled from sperm size. However, transcriptional architecture may play a role through the subfunctionalization of MSP gene expression. In particular, copy number could be correlated with expression level if all genes copies were not equally expressed. In such a scenario, stabilizing selection could act on protein expression level, with gene copy number neutrally evolving. For example, in *Pristionchius* nematodes, gene expression in general is not correlated with lineage-specific duplication events, suggesting that subfunctionalization of copy variants may be common (Baskaran and Rödelsperger 2015). While we annotated multiple MSP genes in each genome, there is currently little to no information outside of *C. elegans* as to whether all gene copies are expressed. While expression data from *C. elegans* show a marginal association of chromosome-level clusters, there is a high degree of variance in expression both within and between clusters. Thus, these existing whole-worm, single developmental stage transcriptome data are too limited to draw any strong conclusions. Important future studies should examine if there is differential expression of copies throughout spermatogenesis and sperm activation. Such a quantitative study of the transcription and translation of MSP genes would be valuable, though challenging due to sequence hyper-conservation.

This neutral model of gene copy expansion seems likely to drive chromosome-level cluster expansion. However, it does not particularly explain the translocation of genes throughout the genome. A distinguishing feature of MSPs is their involvement in reproduction and particularly their function as an oocyte signaling molecule. If pleiotropy constrains the MSP sequence from coevolving with its female receptor, then positive selection may act instead on the gene family to counter any female coevolutionary response. Here, gene conversion could act not only to preserve MSP–MSP interactions, but also to transfer any compensatory mutations due to male–female coevolution to other duplicates (Scienski *et al.* 2015). Adaptive evolution has been shown to drive copy number variation in *C. elegans* on short time scales (Farslow *et al.* 2015) and may explain the dynamic movement of MSP genes throughout the genome of individual lineages, though a direct test of this hypothesis would be challenging. Our study highlights the necessity of using whole-genome data when probing the evolutionary history of a gene. Although the pattern of sequence evolution seen for this reproductive protein is unusual, MSP genes are consistent with a broader perspective in which reproductive interactions are capable of driving rapid evolution at the genome, as well as the sequence, level.

## Supplementary Material

Supplemental material is available online at www.g3journal.org/lookup/suppl/doi:10.1534/g3.117.300281/-/DC1.
