# Supplementary material for: Rapid Gene Family Evolution of a Nematode Sperm Protein Despite Sequence Hyper-conservation
Source: G3 (Bethesda). 2017 Nov 21;8(1):353–62. doi: 10.1534/g3.117.300281 (PMC5765362; doi:10.1534/g3.117.300281)
Supplement: Supplementary file 1 [file 353FigureS1.pdf]

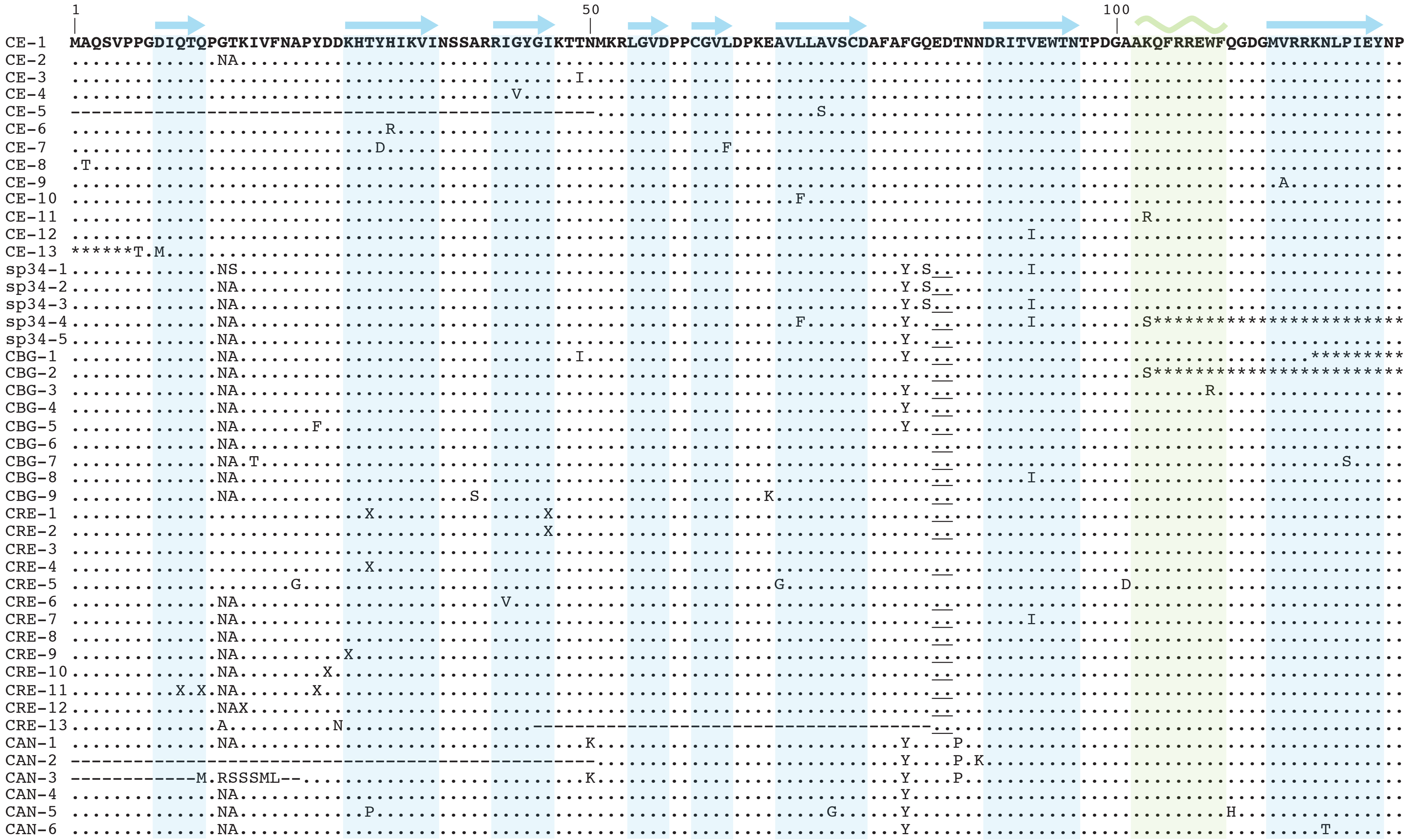

Figure S1. Major sperm protein (MSP) amino acid sequence alignments for the *Caenorhabditis* isopeptide subfamilies (Table S4). Beta sheets are shown in blue and a single alpha helix is shown in green. The underlined residues (83/84) represent the location of the intron splice site. Asterisks denote the position of a diverged N-terminus (CE-13) or C-terminus (sp34-4, CBG-1, and CBG-2). Residues with an X were unable to be translated into an amino acid due to an ambiguous nucleotide in the DNA sequence. Overall, the alignments show the strong conservation of the whole MSP protein sequence.
