# Supplementary material for: Rapid Gene Family Evolution of a Nematode Sperm Protein Despite Sequence Hyper-conservation
Source: G3 (Bethesda). 2017 Nov 21;8(1):353–62. doi: 10.1534/g3.117.300281 (PMC5765362; doi:10.1534/g3.117.300281)
Supplement: Supplementary file 2 [file 353FigureS2.pdf]

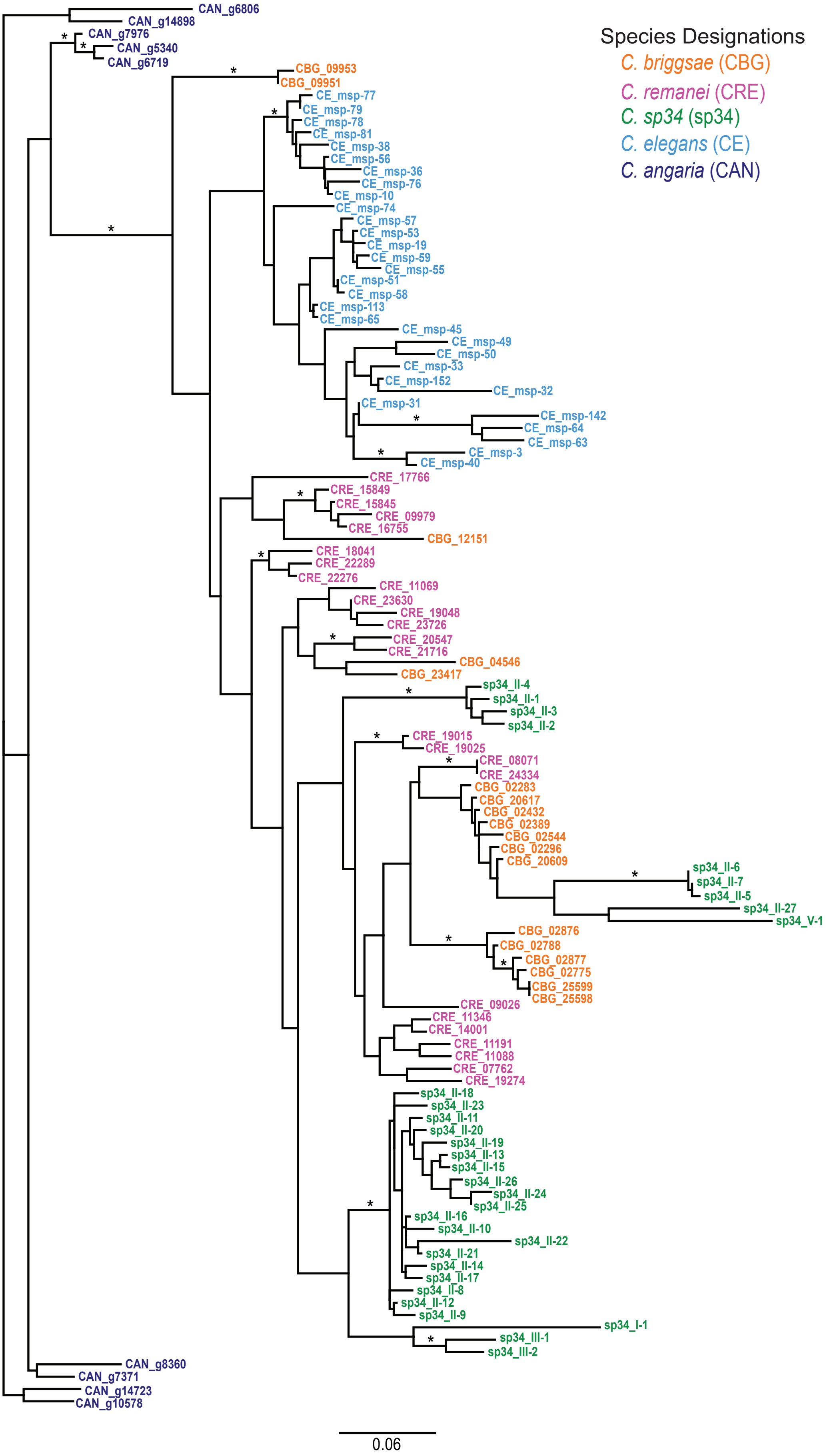

Figure S2. Maximum likelihood phylogeny of all *Caenorhabditis* major sperm protein (MSP) genes. Monophyletic clades correspond to chromosome-level clustering of MSP genes. Despite *C. angaria* falling basally, species relationships are not maintained. Asterisks denote bootstrp values greater than 80%.
