# Supplementary material for: Rapid Gene Family Evolution of a Nematode Sperm Protein Despite Sequence Hyper-conservation
Source: G3 (Bethesda). 2017 Nov 21;8(1):353–62. doi: 10.1534/g3.117.300281 (PMC5765362; doi:10.1534/g3.117.300281)
Supplement: Supplementary file 4 [file 353FileS2.docx]

Table S1. Chromosome II conserved anchors based around *C. elegans* major sperm protein (MSP) genomic positions.

| **Gene** | **Genomic Position** | | | |
| --- | --- | --- | --- | --- |
|  | ***C. elegans*** | ***C. sp. 34*** | ***C. briggsae*** | ***C. remanei*** |
| *ttm-2* | II: 4,764,159 | II: 4,896,552 | II: 10,868,752 | II: 1,876,749 |
| *nep-12* | II: 4,775,526 | II: 4,915,423 | II: 10,853,048 | II: 1,868,427 |
| *msp-3* | II: 4,785,861 |  |  |  |
| *rig-6* | II: 4,798,154 | II: 4,983,564 | II: 10,820,642 | II: 1,841,746 |
| *lact-5* | II: 4,818,662 | II: 5,001,486 | II: 10,813,639 | II: 1,833,803 |
| *msp-40* | II: 4,825,380 |  |  |  |
| *gst-7* | II: 4,882,062 | II: 5,086,328 | II: 2,053,709 | II: 1,796,931 |
| *gst-9* | II: 4,891,573 | II: 5,180, 472 | II: 10,792,051 | II: 1,788,186 |
| *msp-33* | II: 4,892,269 |  |  |  |
| *msp-32* | II: 4,898,616 |  |  |  |
| *btbd-10* | II: 4,905,008 | II: 5,115,785 | II: 10,780,767 | II: 1,782,424 |
| *msp-31* | II: 4,916,179 |  |  |  |
| *msp-152* | II: 4,932,993 |  |  |  |
| *pld-1* | II: 5,084,223 | II: 5,537,837 | II: 10,596,418 | II: 1,641,577 |
| *mpk-2* | II: 5,104,464 | II: 5,606,413 | II: 10,579,609 | II: 1,628,192 |
| *msp-74* | II: 5,115,102 |  |  |  |
| *msp-45* | II: 5,156,700 |  |  |  |
| *msp-49* | II: 5,194,332 |  |  |  |
| *msp-50* | II: 5,199,763 |  |  |  |
| *ampd-1* | II: 5,203,327 | II: 5,806,001 | II: 10,488,040 | II: 1,543,218 |
| *mog-5* | II: 5,399,539 | II: 6,116,098 | II: 10,312,416 | II: 1,391,674 |
| *msp-63* | II: 5,797,065 |  |  |  |
| *msp-142* | II: 5,797,893 |  |  |  |
| *msp-64* | II: 5,809,924 |  |  |  |
| *ptp-2* | II: 5,918,626 | II: 6,923,672 | II: 9,798,108 | II: 963,239 |

Table S2. Chromosome IV major sperm protein genes are unique to *C. elegans*.

| **Gene** | **Genomic Position** | | | |
| --- | --- | --- | --- | --- |
|  | ***C. elegans*** | ***C. sp. 34*** | ***C. briggsae*** | ***C. remanei*** |
| *hot-9* | IV: 5,040,999 | IV^a^ | IV^a^ | IV^a^ |
| *msp-55* | IV: 5,055,593 |  |  |  |
| *msp-57* | IV: 5,062,543 |  |  |  |
| *msp-53* | IV: 5,064,641 |  |  |  |
| *soc-2* | IV: 5,127,006 | IV^a^ | IV^a^ | IV^a^ |
| *ttbk-2* | IV: 5,258,685 | IV^a^ | I^a^ | 154^a^ |
| *msp-19* | IV: 5,260,554 |  |  |  |
| *nlp-47* | IV: 5,290,847 | IV^a^ | – | IV^a^ |
| *msp-51* | IV: 5,294,922 |  |  |  |
| *msp-113* | IV: 5,297,927 |  |  |  |
| *msp-58* | IV: 5,308,593 |  |  |  |
| *msp-59* | IV: 5,312,690 |  |  |  |
| *msp-65* | IV: 5,316,488 |  |  |  |
| *gba-4* | IV: 5,344,633 | IV^a^ | IV^a^ | IV^a^ |
| *rme-2* | IV: 5,469,995 | III | IV^a^ | 655^a^ |
| *ins-7* | IV: 9,682,013 | – | – | – |
| *msp-76* | IV: 9,688,982 |  |  |  |
| *ssp-36* | IV: 9,765,706 | III | III | IV^a^ |
| *msp-79* | IV: 9,766,745 |  |  |  |
| *msp-78* | IV: 9,770,030 |  |  |  |
| *cav-1* | IV: 9,772,510 | IV^a^ | IV^a^ | IV^a^ |
| *msp-81* | IV: 9,835,997 |  |  |  |
| *msp-10* | IV: 9,837,632 |  |  |  |
| *msp-56* | IV: 9,841,443 |  |  |  |
| *sss-1* | IV: 9,892,769 | IV^a^ | – | 37^a^ |
| *msp-77* | IV: 9,894,596 |  |  |  |
| *ssp-32* | IV: 9,895,418 | III | – | 262^a^ |
| *ttr-39* | IV: 10,088,483 | IV^a^ | IV^a^ | IV^a^ |
| *msp-36* | IV: 10,095,400 |  |  |  |
| *dis-3* | IV: 10,097,208 | IV^a^ | IV^a^ | IV^a^ |
| *gst-4* | IV: 10,142,240 | II | IV^a^ | IV^a^ |
| *msp-38* | IV: 10,143,312 |  |  |  |
| *msp-37* | IV: 10,143,995 |  |  |  |
| *tsp-12* | IV: 10,149,163 | IV^a^ | IV^a^ | IV^a^ |

^a^Chromosomes or scaffolds to which no MSP genes mapped.

Table S3. The evolution of the actin gene family in *Caenorhabditis*. Sequence divergence is given as the median number of pairwise amino acid residue changes between the annotated actin copies of each species and the paralogous *C. elegans* reference gene(s). The lower and upper quartiles of the pairwise divergences are given in parentheses.

| **Paralogs** | **Species** | **Gene (Position)** | **Median Residue Changes** |
| --- | --- | --- | --- |
| 1, 2, 3 | *C. elegans* | *act-1* (V: 11,079,167)  *act-2* (V: 11,076,723)  *act-3* (V: 11,071,690) | 3 (1, 29.3) |
|  | *C. sp. 34* | Sp34_actV-1 (V: 16,628,033)  Sp34_actV-2 (V: 16,634,712)  Sp34_actV-3 (V: 16,637,678) |  |
|  | *C. remanei* | FL81_09400 (V: 1,034,367)  FL81_09406 (V: 1,045,077) |  |
|  | *C. briggsae* | CBG_*act-2* (V: 9,981,924)  CBG_23090 (V: 9,986,779)  CBG_24444 (un: 1,384,354) |  |
|  | *C. angaria* | – |  |
| 4 | *C. elegans* | *act-4* (X: 4,960,553) | 3 (3, 7.8) |
|  | *C. sp. 34* | Sp34_actX-1 (X: 16,364,976) |  |
|  | *C. remanei* | FL81_02266 (X: 11,190,941) |  |
|  | *C. briggsae* | CBG_02165 (X: 4,607,858) |  |
|  | *C. angaria* | CAN_13387 (680: 28,681) |  |
| 5 | *C. elegans* | *act-5* (III: 13,604,339) | 4 (2, 7.5) |
|  | *C. sp. 34* | Sp34_actIII-1 (III: 6,712,969) |  |
|  | *C. remanei* | FL81_20064 (127: 193,874) |  |
|  | *C. briggsae* | CBG_*act-5* (III: 5,511,700) |  |
|  | *C. angaria* | CAN_7888 (250: 70,972) |  |

Table S4. Species-specific major sperm protein subfamilies based on identical peptide sequence.

| **Subfamily** | **Gene Annotation Number** | **Genomic Position** | **Protein Length** |
| --- | --- | --- | --- |
| CE-1 | msp-19 | IV: 5,260,554 | 127 |
|  | msp-31 | II: 4,916,179 | 127 |
|  | msp-40 | II: 4,825,380 | 127 |
|  | msp-45 | II: 5,156,700 | 127 |
|  | msp-50 | II: 5,199,763 | 127 |
|  | msp-51 | IV: 5,294,922 | 127 |
|  | msp-53 | IV: 5,064,641 | 127 |
|  | msp-59 | IV: 5,312,690 | 127 |
|  | msp-64 | II: 5,809,924 | 127 |
|  | msp-65 | IV: 5,316,488 | 127 |
|  | msp-74 | II: 5,115,102 | 77 |
|  | msp-81 | IV: 9,835,997 | 127 |
|  | msp-113 | IV: 5,297,927 | 127 |
|  | msp-142 | II: 5,797,893 | 127 |
| CE-2 | msp-10 | IV: 9,937,632 | 127 |
|  | msp-36 | IV: 10,095,400 | 127 |
|  | msp-56 | IV: 9,841,443 | 127 |
|  | msp-76 | IV: 9,688,982 | 127 |
| CE-3 | msp-49 | II: 5,194,332 | 127 |
| CE-4 | msp-55 | IV: 5,055,593 | 127 |
|  | msp-57 | IV: 5,062,543 | 127 |
| CE-5 | msp-58 | IV: 5,308,593 | 77 |
| CE-6 | msp-63 | II: 5,797,065 | 127 |
| CE-7 | msp-38 | IV: 10,143,312 | 127 |
| CE-8 | msp-152 | II: 4,932,993 | 127 |
| CE-9 | msp-77 | IV: 9,766,745 | 127 |
|  | msp-79 | IV: 9,770,030 | 127 |
| CE-10 | msp-33 | II: 4,892,269 | 127 |
| CE-11 | msp-3 | II: 4,785,861 | 127 |
| CE-12 | msp-78 | IV: 9,770,030 | 127 |
| CE-13 | msp-32 | II: 4,898,616 | 190 |
| Sp34-1 | Sp34_II-24 | II: 11,949,545 | 127 |
|  | Sp34_II-25 | II: 11,950,759 | 127 |
| Sp34-2 | Sp34_II-11 | II: 10,887,101 | 127 |
|  | Sp34_II-19 | II: 11,118,785 | 127 |
|  | Sp34_II-20 | II: 11,129,314 | 127 |
| Sp34-3 | Sp34_II-13 | II: 11,026,157 | 127 |
|  | Sp34_II-15 | II: 11,045,299 | 127 |
|  | Sp34_II-26 | II: 11,963,975 | 127 |
| Sp34-4 | Sp34_II-22 | II: 11,143,891 | 127 |

Table S4 *continued*

| **Subfamily** | **Gene Annotation Number** | **Genomic Position** | **Protein Length** |
| --- | --- | --- | --- |
| Sp34-5 | Sp34_II-1 | II: 3,978,246 | 127 |
|  | Sp34_II-2 | II: 4,865,714 | 127 |
|  | Sp34_II-3 | II: 4,938,892 | 127 |
|  | Sp34_II-4 | II: 5,191,848 | 127 |
|  | Sp34_II-5 | II: 7,331,314 | 127 |
|  | Sp34_II-6 | II: 7,339,167 | 127 |
|  | Sp34_II-7 | II: 7,354,781 | 127 |
|  | Sp34_II-8 | II: 10,878,671 | 127 |
|  | Sp34_II-9 | II: 10,880,233 | 127 |
|  | Sp34_II-10 | II: 10,884,043 | 127 |
|  | Sp34_II-12 | II: 10,901,031 | 127 |
|  | Sp34_II-14 | II: 11,027,906 | 127 |
|  | Sp34_II-16 | II: 11,108,284 | 127 |
|  | Sp34_II-17 | II: 11,115,902 | 127 |
|  | Sp34_II-18 | II: 11,118,068 | 127 |
|  | Sp34_II-21 | II: 11,142,848 | 127 |
|  | Sp34_II-23 | II: 11,561,823 | 127 |
|  | Sp34_II-27 | II: 18,759,892 | 127 |
|  | Sp34_V-1 | V: 18,527,789 | 127 |
|  | Sp34_I-1 | I: 16,294,742 | 127 |
|  | Sp34_III-1 | III: 1,490,273 | 127 |
|  | Sp34_III-2 | III: 1,584,672 | 127 |
| CBG-1 | CBG09951 | III: 9,157,912 | 154 |
| CBG-2 | CBG02788 | II: 8,699,198 | 134 |
| CBG-3 | CBG02544 | II: 9,768,091 | 127 |
| CBG-4 | CBG02432 | II: 10,178,939 | 127 |
|  | CBG02283 | II: 10,734,729 | 127 |
|  | CBG02296 | II: 10,682,304 | 127 |
|  | CBG09953 | III: 9,170,158 | 127 |
|  | CBG20609 | II: 10,848,018 | 127 |
|  | CBG20617 | II: 10,875,038 | 127 |
| CBG-5 | CBG02389 | II: 10,309,523 | 127 |
| CBG-6 | CBG02775 | II: 8,740,254 | 127 |
|  | CBG02877 | II: 8,341,259 | 127 |
|  | CBG12151 | V: 5,396,316 | 127 |
|  | CBG25598 | II: 8,626,424 | 127 |
|  | CBG25599 | II: 8,635,477 | 127 |
| CBG-7 | CBG04546 | V: 14,368,719 | 127 |
| CBG-8 | CBG23417 | V: 8,752,470 | 127 |
| CBG-9 | CBG02876 | II: 8,343,124 | 127 |
| CRE-1 | FL81_22289 | 198: 73,491 | 127 |
| CRE-2 | FL81_18041 | 72: 44,488 | 127 |
| CRE-3 | FL81_17766 | 76: 112,358 | 127 |

Table S4 *continued*

| **Subfamily** | **Gene Annotation Number** | **Genomic Position** | **Protein Length** |
| --- | --- | --- | --- |
| CRE-4 | FL81_15845 | 42: 26,874 | 127 |
|  | FL81_16755 | 55: 38,4833 | 127 |
|  | FL81_22276 | 198: 29,841 | 127 |
| CRE-5 | FL81_09979 | 5: 496,198 | 127 |
| CRE-6 | FL81_11346 | II: 1,760,482 | 127 |
| CRE-7 | FL81_09026 | II: 12,083,855 | 127 |
|  | FL81_19015 | 91: 272,019 | 77 |
|  | FL81_19025 | 91: 306,535 | 77 |
|  | FL81_20547 | 135: 17,675 | 127 |
| CRE-8 | FL81_07762 | II: 5,309,307 | 127 |
|  | FL81_08071 | II: 6,657,573 | 127 |
|  | FL81_11088 | II: 722,956 | 127 |
|  | FL81_15849 | 42: 36,489 | 77 |
|  | FL81_21716 | 197:18,758 | 127 |
|  | FL81_23630 | 318: 72,252 | 127 |
|  | FL81_23726 | 361: 13,450 | 127 |
|  | FL81_24334 | 446: 42,294 | 127 |
| CRE-9 | FL81_11069 | II: 676,158 | 127 |
| CRE-10 | FL81_11191 | II: 1,181,817 | 127 |
| CRE-11 | FL81_19274 | 90: 90,830 | 127 |
| CRE-12 | FL81_19048 | 99: 58,251 | 127 |
| CRE-13 | FL81_14001 | 17: 807,783 | 89 |
| CAN-1 | g6719 | 194: 97,280 | 127 |
| CAN-2 | g5340 | 136: 111,178 | 77 |
| CAN-3 | g256 | 256: 13,580 | 113 |
| CAN-4 | g6806 | 198: 102,254 | 127 |
|  | g7371 | 225: 56,927 | 127 |
|  | g14723 | 881: 17,664 | 127 |
|  | g14898 | 913: 16,835 | 127 |
| CAN-5 | g8360 | 278: 37,278 | 127 |
| CAN-6 | g10578 | 418: 21,710 | 127 |
